# Comprehensive early warning strategies based on consistency deviation of thermal–electrical characteristics for energy storage grid

**DOI:** 10.1016/j.isci.2021.103058

**Published:** 2021-08-28

**Authors:** Xiaogang Wu, Zhihao Cui, Gang Zhou, Tao Wen, Fangfang Hu, Jiuyu Du, Minggao Ouyang

**Affiliations:** 1 School of Electrical and Electronic Engineering, Harbin University of Science and Technology, Harbin 150080, China; 2 State Key Laboratory of Automotive Safety and Energy, Tsinghua University, Beijing 100084, China; 3 State Grid Fujian Electric Power CO., Ltd, Fuzhou 350003, China; 4 Beijing Products Quality Supervision and Inspection Institute, Beijing 101300, China

**Keywords:** energy resources, energy systems, energy management, energy storage

## Abstract

Lithium iron phosphate (LiFePO_4_) batteries have been dominant in energy storage systems. However, it is difficult to estimate the state of charge (SOC) and safety early warning of the batteries. To solve these problems, this paper developed a multiple timescale comprehensive early warning strategy based on the consistency deviation of the electrical and thermal characteristics of LiFePO_4_ batteries. The unscented Kalman filter method was employed to estimate the battery SOC. The established comprehensive early warning strategy was verified through fault-triggered experiments at different time scales with different equivalent resistances. The results show that the comprehensive early warning strategy can realize early warning for different timescale failures of LiFePO_4_ batteries under different energy storage conditions. For more dangerous severe failures that can break the safety valve, safety early warning can be realized 15 min in advance. This study provides a reference to ensure safe and reliable operations of energy storage systems.

## Highlights

• A comprehensive early warning strategy for multiple timescales was developed
• The battery electric-thermal characteristics of different time scales were obtained
• The proposed warning strategy expanded the application of early warning algorithms

## Graphical abstract

**Figure:**
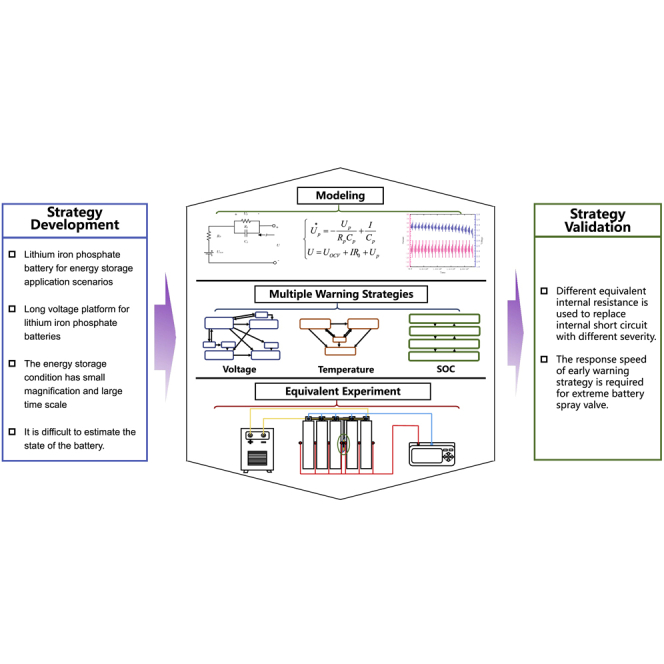

## Introduction

Renewable energy technology has been widely employed in power generation systems due to its low-carbon emission and environmental friendliness. However, due to the instability of renewable energy generation, such as wind and solar energy, the application of energy storage systems is indispensable in renewable energy generation systems. Lithium iron phosphate (LiFePO_4_) batteries are widely used in energy storage power stations due to their long life and high energy and power densities (Lu et al., 2013; Han et al., 2019). However, frequent fire accidents in energy storage power stations have induced anxiety about the safety of large-scale lithium-ion (Li-ion) battery systems. In 2019, a fire explosion occurred in the 2.47-MWh lithium battery system in Arizona, USA. The final investigation report proved that the fire was caused by the internal defects in the batteries, especially the formation of abnormal lithium dendrites (Hill, 2020). Thermal runaway is a major concern in the large-scale application of Li-ion batteries (Ren et al., 2019). There is an urgent need for effective safety early warning management for Li-ion batteries during operations.

Studies on safety early warning in Li-ion batteries have employed consistency differences of batteries in a module for fault diagnosis. Ouyang et al. (Ouyang et al., 2015) used an equivalent circuit model to analyze the electrical characteristics of an internal short circuit in a large Li-ion battery. They proposed an internal short-circuit detection method based on the consistency of batteries. This internal short-circuit detection method employs the recursive least-square algorithm to calculate the characteristic parameters, such as the voltage difference and the fluctuation function of the internal resistance. Internal short-circuit detection is realized through a change in the characteristic parameters. Feng et al. (Feng et al., 2016) proposed an online detection method for internal short circuits, which achieves online detection of internal short circuits through model parameterization and parameter estimation. Using a three-dimensional (3D) electrical–thermal internal short-circuit coupling model, they discussed the correlation between the measured voltage, current, and temperature data and the internal short-circuit state. Gao et al. (Gao et al., 2019) conducted a quantitative analysis of micro-internal short circuits in the initial stage. Based on the battery state of charge (SOC) difference model, the extended Kalman filter was used to estimate the difference between the battery pack and average SOC. On this basis, a microinternal short-circuit analysis based on recursive least squares was proposed. Kong et al. (Kong et al., 2018) proposed an analysis method for microinternal short circuits based on the change in the remaining charge capacity among batteries. Based on the voltage curve of the first fully charged battery in the module, the remaining charging capacity of each battery was obtained by converting the voltage curve. Internal short circuits were identified by an increase in the remaining charge capacity after each charge. Zhang et al. (Zhang et al., 2019) proposed a method for identifying micro short circuits under dynamic conditions. Similar electric quantity differences were obtained through the open-circuit voltage (OCV) differences and data smoothing function of the low-pass filter. The short-circuit current and internal resistance were estimated using the change in electric quantity difference and verified through experiments on the actual battery pack. Meanwhile, some studies started with fault data and conducted safety early warning and fault identification in Li-ion batteries through the rules of fault data (Gao et al., 2020; Kang et al., 2020; Naha et al., 2020). Battery fault analyses using new sensors have also been reported. Cai et al. (Cai et al., 2021) used nondispersive infrared (NDIR) CO_2_ sensor to detect vent-gas and battery failure. An overcharging experiment leading to cell venting was conducted using a prototype gas sensor suite. However, most existing studies focused on vehicle operating conditions and high-energy-density ternary Li-ion batteries (Tomaszewska et al., 2019). Studies on LiFePO_4_ batteries commonly used in energy storage systems are relatively few, and the operating conditions of energy storage systems have rarely been studied.

LiFePO_4_ batteries have higher safety than ternary Li-ion batteries due to the nature of the cathode material (Bugryniec et al., 2019). However, due to the presence of an electrolyte, LiFePO_4_ batteries still have the risk of thermal runaway. Considering state estimation, due to the OCV plateau in the voltage curve and the hysteresis phenomenon in LiFePO_4_ batteries (Roscher et al., 2011), it is very difficult to accurately estimate the SOC of LiFePO_4_ batteries. Especially for safety early warning applications, due to the short-circuit current, the commonly used ampere-hour integration method cannot accurately estimate the SOC of the batteries. They only rely on the terminal voltage change in the batteries to correct the SOC estimation error. Thus, the safety early warning of LiFePO_4_ batteries in energy storage systems is difficult.

To address the problem of safety early warning in LiFePO_4_ batteries in energy storage systems, we propose a multitime scale comprehensive early warning strategy based on the consistency deviation of electric and thermal characteristics. The observed values of consistency deviation of voltage, temperature, and SOC are selected as the characteristic parameters for the safety early warning strategy. The unscented Kalman filter (UKF) method is used to estimate the SOC of LiFePO_4_ batteries, and the heat-generation internal resistance is estimated using the recursive least-square method. Early warning strategies are formulated based on comprehensive decision-making and several characteristic information. The effectiveness of the proposed comprehensive early warning strategy is verified through different equivalent internal-resistance fault-trigger experiments, which simulate the failure of energy storage battery modules at different time scales. The type of fault simulated is an internal short circuit fault of the battery. In the late stage of an internal short circuit fault, in addition to extremely fast power dissipation, it is accompanied by severe heat production, and when the battery temperature exceeds its thermal runaway trigger temperature, the battery will enter a thermal runaway state. Severe thermal runaway can lead to the battery internal material from the drain valve ejected or even fire. It is generally believed that the thermal runaway process of the battery often has an internal short circuit as a hallmark feature. Therefore, in order to improve the safety of the energy storage system, it is necessary to provide early warning of the internal short circuit failure of the battery to prevent the development of the internal short circuit failure of the battery to a late stage, and the results of the safety warning can provide a quantitative basis for the safety maintenance and emergency disposal of the energy storage system. In this paper, a comprehensive warning strategy based on consistency deviation is developed for energy storage application scenarios, which can achieve early warning for different time scales of lithium iron phosphate battery failures under energy storage conditions, and can warn more than 15 min in advance for serious failures that can lead to battery valve injection, which meets the time margin requirement for safety warning in energy storage scenarios.

**Figure 1:**
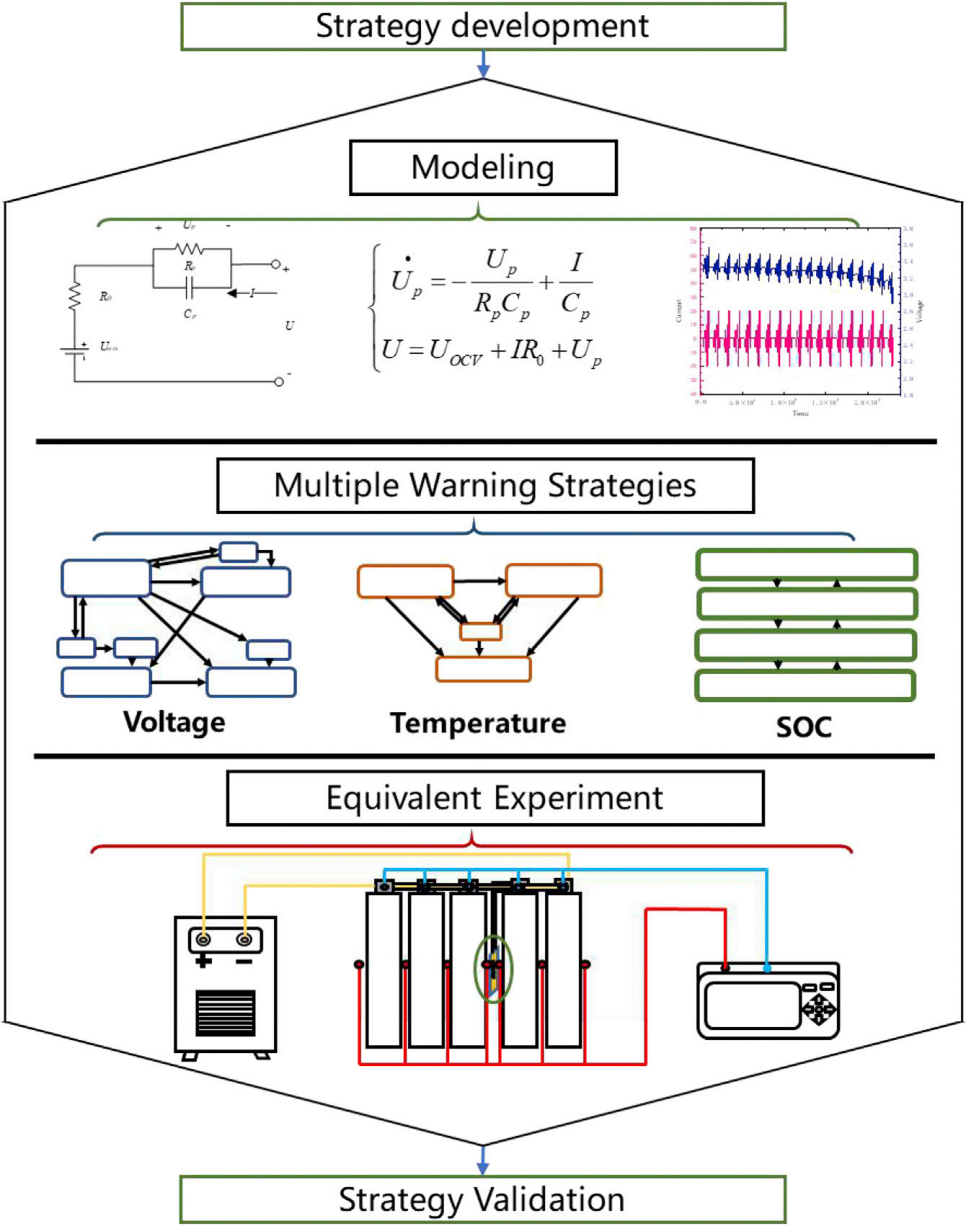
Flowchart of this study

Figure 1 shows a flowchart of this study. The equivalent circuit model of LiFePO_4_ batteries is first established. Based on the equivalent circuit model, the state estimation algorithm and early warning strategy were developed. Then, the equivalent internal resistance values of 5, 1, and 0.05 Ω were used to trigger the equivalent substitution experiment. The failure of the energy storage battery with multiple time scales was simulated. The fault data for different time scales were obtained. The early warning strategy was verified and analyzed through the fault data.

## Equivalent circuit modeling of battery

Herein, the consistency deviation of battery state and the estimation of battery SOC based on UKF are employed for fault diagnosis. The reason for choosing UKF is that compared to NCM batteries, LiFePO4 batteries have a flatter voltage curve, which will pose a great challenge to the SOC estimation of the battery. The EKF with first-order theoretical accuracy does not meet the requirements of the early warning algorithm for state estimation accuracy. In contrast, UKF is a method of determining samples through a traceless transformation, capturing the mean and covariance of Gaussian random variables with fewer samples, and its theoretical accuracy can reach third order and above, so we adopt UKF as the SOC estimation algorithm in the early warning algorithm. This method does not rely on existing fault data but recognizing the consistency of the battery status between battery packs. To estimate the SOC of a battery using the early warning strategy, there is a need for the equivalent circuit model of the battery and to calibrate the parameters of the model. The equipment used in this research is as follows: the charging and discharging equipment is Digatron BTS-600; the data acquisition device is Hioki LR8450; the temperature setting value of the temperature chamber is 25°C during the experiments.

### Basic parameters of batteries

Herein, we consider a 20-Ah LiFePO_4_ battery, which is widely used in energy storage systems. The specific parameters of the battery are listed in Table 1.

**Table 1:** Battery specification

Parameters (unit)	Numerical value
Capacity (Ah)	20 (measured: 21.8)
Nominal voltage (V)	3.2
Positive and negative materials	LiFePO_4_/graphite
DC internal resistance (mΩ)	≤6

### OCV test

**Figure 2:**
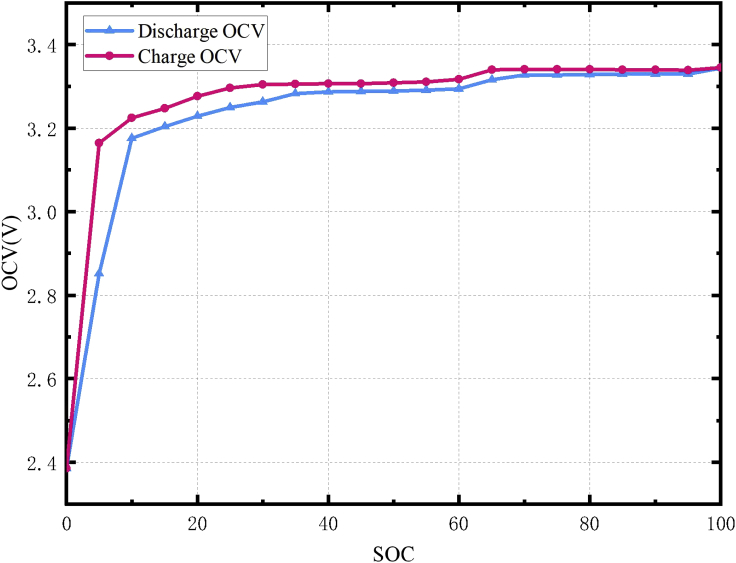
OCV–SOC curves of a LiFePO_4_ battery

The OCV of a Li-ion battery refers to the terminal voltage of the battery after a long enough period of rest, which is determined by the material properties of the positive and negative electrodes of the battery. Due to the hysteresis characteristics of LiFePO_4_ batteries, the OCV curve of the battery during the charging process does not coincide with that of the discharge process. Therefore, the OCV of the battery is measured separately for the charging and discharging process (Roscher and Sauer, 2011). The specific test procedure is as follows. A charge rate of 0.5 C (1 C rate corresponds to a charge current of 20 A, 0.5 C is 10 A) is used to charge a battery with an SOC of 0%. The battery is charged with a capacity of 5% SOC each time until the battery is fully charged. After charging, the battery rest for 3 h for OCV measurements. Then, the battery is discharged. The OCV–SOC curves of a LiFePO_4_ battery measured through this experiment are shown in Figure 2.

The OCV–SOC curve of the LiFePO_4_ battery has a strong nonlinear relationship. There are two very flat voltage plateaus at SOC of 30%–60% and 70%–90%, where the OCV of the battery changes very little with the SOC. A maximum OCV change of 1 mV could result in a 5% SOC change (Zheng et al., 2013). This makes it difficult to estimate the SOC of LiFePO_4_ batteries. Moreover, the hysteresis phenomenon causes the OCV–SOC curve to vary during the charging and discharging process, and the hysteresis voltage can be more than 50 mV. Herein, the energy storage conditions used are relatively complicated because there is no complete charging process, which makes it difficult to correct the SOC of the battery. It is difficult to consider the effect of hysteresis when the historical SOC is unknown. Therefore, the OCV of the battery is selected as the average value of the OCV of the battery charging and discharging process. The final estimation results also prove its effectiveness.

### Hybrid pulse power characteristic test

**Figure 3:**
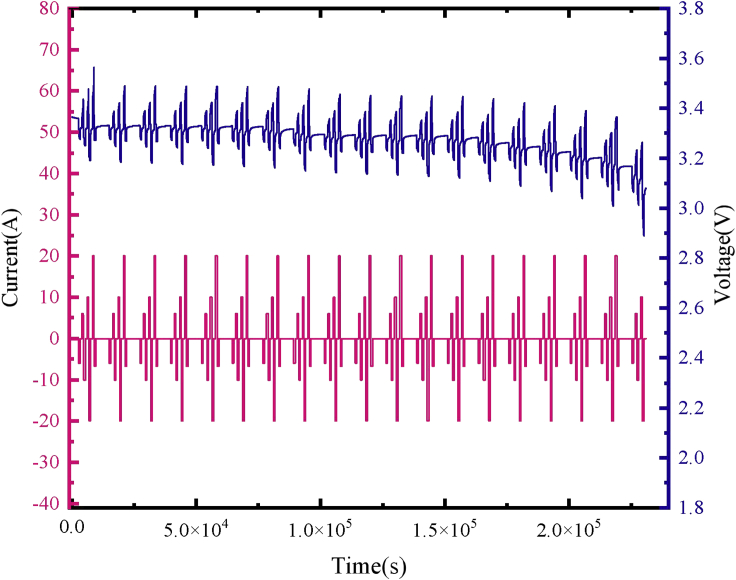
HPPC test result

Hybrid pulse power characteristic (HPPC) is a widely used performance test for Li-ion batteries. A short-time charge/discharge pulse is used to test the performance of batteries. The common test standard is to apply a 1.0-C pulse current consisting of 10-s discharge pulse, 40-s rest, and 10-s charging pulse to the battery every 5% SOC to observe the voltage response. Herein, according to the requirements of energy storage conditions, the charging and discharging pulse is extended to 60 s, which is closer to the frequency of the current profile in energy storage. Simultaneously, the pulse amplitudes of 0.3, 0.5, and 1.0 C are measured. The final measurement result is shown in Figure 3. To improve the accuracy of the HPPC test results, the sampling interval is changed from 1.0 to 0.1 s to achieve a better sampling effect when the test is conducted in the pulse section.

### Thevenin equivalent circuit model

**Figure 4:**
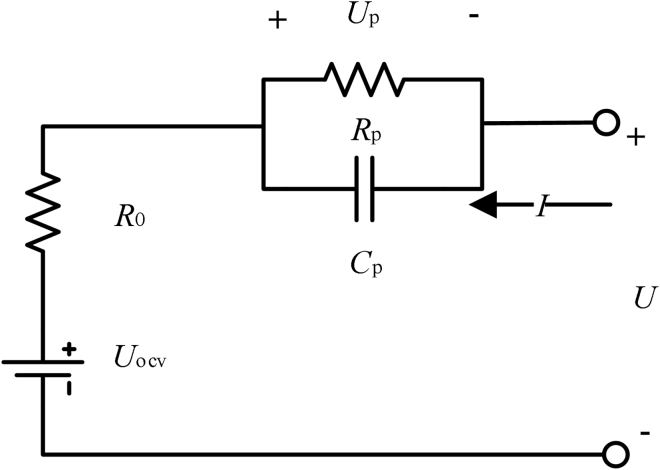
The venin equivalent circuit model

Herein, One-order Thevenin equivalent circuit model is used to analyze the safety early warning strategy and estimate the SOC (Hu et al., 2012). This model is widely used in the engineering field to estimate SOC. Although the higher-order equivalent circuit model has higher model accuracy, the significance of its model parameters is more reflected in the internal electrochemical properties of the battery. For the application scenario of battery SOC estimation under energy storage conditions, the accuracy of the first-order equivalent circuit model has satisfied the need of the early warning algorithm for SOC estimation accuracy. As shown in Figure 4, the one-order Thevenin equivalent model consists of battery OCV (*U*_OCV_), Ohm resistance *R*_0_, polarization resistance *R*_p_, and polarization capacitance *C*_p_. The current flowing into the battery is selected as the positive reference direction, and the model can be expressed by Equation (1):

(Equation 1) $$\left\{ \begin{array}{l} {\overset{\cdot }{U}}_{\text{p}}=-\frac{U_{\text{p}}}{R_{\text{p}}C_{\text{p}}}+\frac{I}{C_{\text{p}}}\\ U=U_{\text{OCV}}+IR_{0}+U_{\text{p}} \end{array}\right.$$

The parameters in the equivalent circuit model are generally obtained through the performance test of the battery and are realized by parameter identification. Herein, because the early warning strategy is designed for energy storage application, the working temperature of the battery is relatively stable. Therefore, the performance test and parameter identification of the Thevenin equivalent circuit model of the battery are based on 25°C.

### Parameter identification based on genetic algorithm

According to the Thevenin equivalent circuit model, the terminal voltage and current of the battery can be calculated as follows:

(Equation 2) $$U_{\text{P}}\left(k\right)=e^{-\frac{\Delta t}{R_{\text{p}}C_{\text{p}}}}\cdot U_{\text{P}}\left(k-1\right)+I\left(k\right)\cdot R_{\text{p}}\left(1-e^{-\frac{\Delta t}{R_{\text{p}}C_{\text{p}}}}\right),$$

(Equation 3) $$U_{model}\left(k\right)=U_{\text{OCV}}\left(k\right)+U_{\text{p}}\left(k\right)+I\left(k\right)\cdot R_{\text{0}},$$

where *k* is the discrete time points, and Δ*t* is the sampling interval. The ohmic internal resistance *R*_0_ can be directly calculated from the voltage response of the battery at the beginning of the charging and discharging pulse using Ohm's law as follows:

(Equation 4) $$R_{\text{cha/dch}}=\frac{U_{0.1s}-U_{0s}}{I_{\text{pulse}}},$$

where *R*_cha/dch_ represents the ohmic internal resistance of the battery during the charging and discharging process, *U*_0s_ and *U*_0.1s_ are the battery terminal voltage before the pulse and 0.1 s after the start of the pulse, respectively, and *I*_pulse_ is the amplitude of the pulse current.

**Figure 5:**
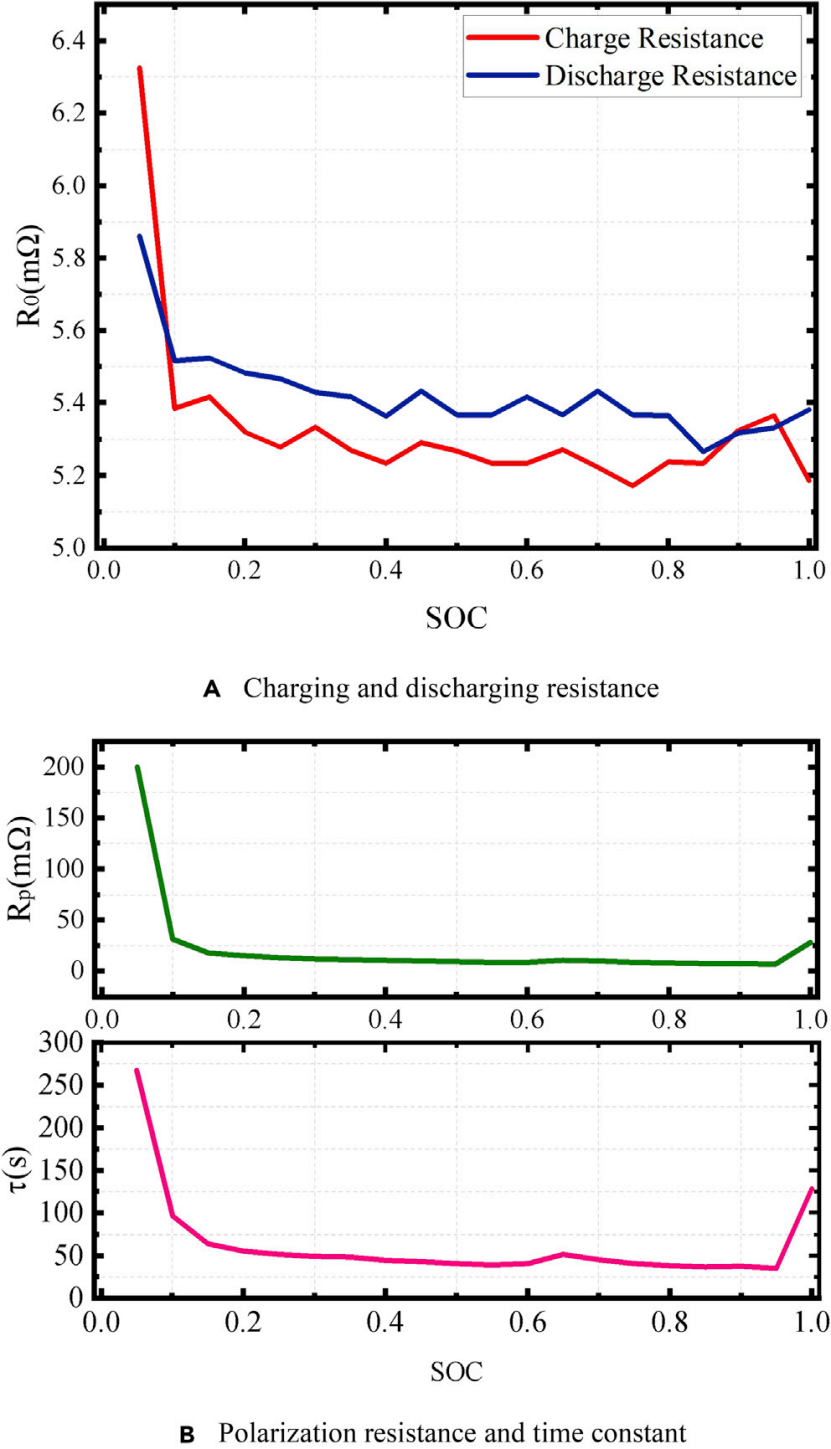
Parameters of equivalent circuit model (A) Charging and discharging resistance. (B)Polarization resistance and time constant.

The polarization resistance and capacitance, *R*_p_ and *C*_p_, respectively, can be optimized using a genetic algorithm. Considering the root-mean-square error of the battery terminal voltage model obtained from the model and experiment as the optimization objective, *R*_p_ and *C*_p_ of the battery that minimize the optimization objective can be calculated. Herein, the population size of the genetic algorithm is 100, and the genetic algebra is 100 generations. The parameters of the equivalent circuit model optimized by the genetic algorithm are shown in Figure 5. Time constant τ is employed to be more intuitive (τ = *R*_p_*C*_p_). The ohmic resistance, *R*_p_, and *C*_p_ of the battery increase significantly in the low-SOC range.

## Safety early warning strategy of LiFePO_4_ batteries based on the consistent deviation of electrical and thermal characteristics

Herein, the safety-warning strategy is realized by calculating the consistency deviation of the electrical and thermal characteristics of the battery. To distinguish different time scales and fault levels, the battery fault states are divided into normal, slightly fault, medium fault, and serious fault states. Then, a safety early warning strategy based on the consistency deviation of voltage, temperature, and SOC is determined. The state transfer parameters are calibrated by the pattern of changes in the electrical, thermal, and SOC of the cell during the short-circuit substitution experiments within the module. When a serious fault occurs, the safety-warning strategy sends an warning signal.

### Safety early warning strategy based on voltage consistency deviation

**Figure 6:**
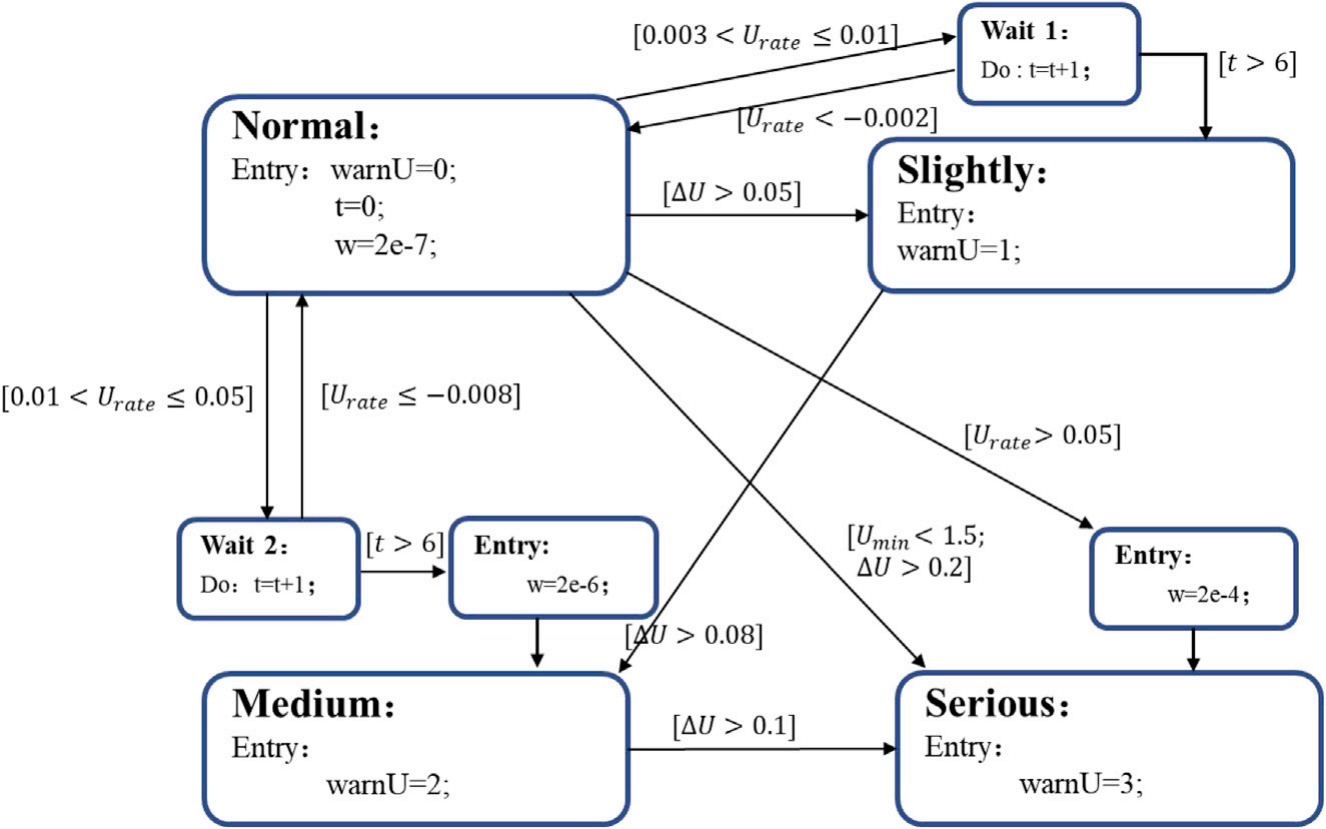
Safety early warning strategy based on voltage consistency

Safety early warning strategy based on the deviation in voltage consistency is shown in Figure 6. The voltage difference Δ*U*, change rate of voltage difference *U*_rate_, and the lowest battery terminal voltage in the module *U*_min_ are used as warning characteristic parameters. WarnU is the voltage fault signal corresponding to 1, 2, and 3 in slightly fault state, medium fault state, and serious fault state, respectively, and *w* is a parameter in the UKF algorithm.

Voltage difference Δ*U* refers to the voltage difference between the average and lowest voltages, *U*_mean_ and *U*_min_, respectively. The thresholds of the voltage difference Δ*U* are set as 0.05, 0.08, and 0.1 V. When the voltage difference reaches a threshold, the fault state changes. A fast-rising path is set for serious fault states when Δ*U* is greater than 0.2 V. Then, serious failure occurs, and the state can directly change from normal state to serious state. The change rate of voltage difference refers to the change rate of Δ*U*. This method can avoid the influence of poor consistency in the battery pack to a certain degree and can prevent instantaneous voltage change rate interference caused by the sudden change in current excitation applied to the battery. Specifically, *U*_rate_ can be obtained by differentiating the voltage difference of the battery as follows:

(Equation 5) $$U_{\text{rate}}=\frac{d\Delta U}{dt}=\frac{d\left(U_{\text{mean}}-U_{\text{min}}\right)}{dt}$$

**Figure 7:**
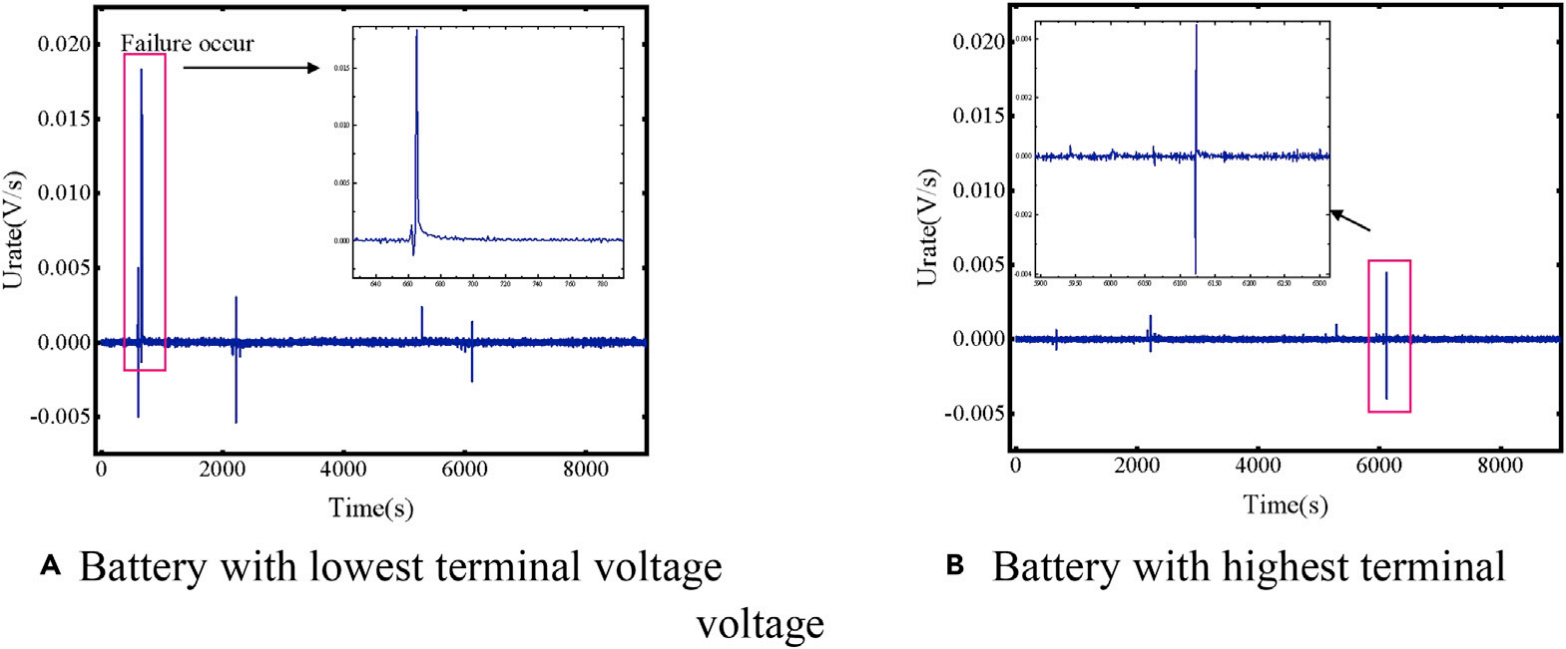
Battery rate of change in voltage difference from module experiments with an equivalent internal resistance of 1 Ω (A) Battery with lowest terminal voltage. (B) Battery with highest terminal voltage.

We set the thresholds of *U*_rate_ to be 0.003, 0.01, and 0.05 V/s. When *U*_rate_ is greater than the thresholds, the state changes. When the current profile changes and the voltage sampling are not synchronized, a relatively high *U*_rate_ may occur, leading to a false warning signal. As shown in Figure 7, due to the asynchronous voltage sampling, there is also a high rate of change in voltage difference besides the time that failure occurs. However, synchronization is usually completed within a few seconds, after which a negative *U*_rate_ of similar amplitudes appears. Therefore, a waiting state with a duration of 6 s is set to prevent the occurrence of false warning signals. When a reverse *U*_rate_ with a similar amplitude is detected in the waiting state, the state returns to the normal state and avoids issuing early warning signals.

Considering the battery working area, the threshold of the early warning strategy with the lowest battery terminal voltage is set as 1.5 V. When the lowest battery voltage in the module *U*_min_ is lower than 1.5 V, the state of the early warning strategy changes directly to the serious fault state.

### Safety early warning strategy based on temperature consistency deviation

**Figure 8:**
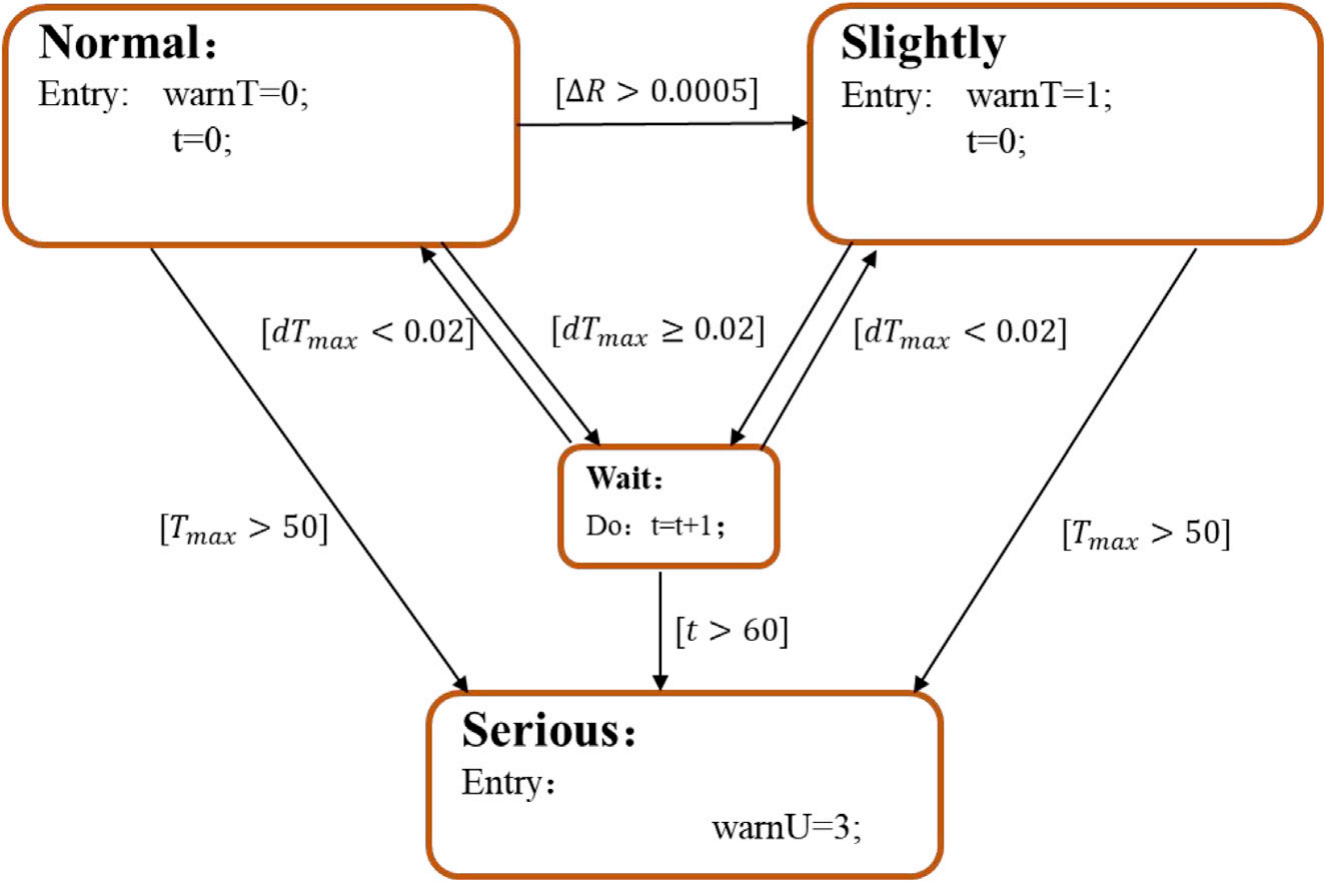
Safety early warning strategy based on temperature consistency deviation

The safety early warning strategy based on temperature consistency deviation is shown in Figure 8. This strategy takes the highest battery temperature in the module *T*_max_, maximum rate of change in temperature d*T*_max_, and difference in heat-generating internal resistance Δ*R* as early warning characteristic parameters. When *T*_max_ reaches the threshold of 50°C, or d*T*_max_ exceeds the threshold of 0.02°C/s for 60 s, it is considered that the battery has obvious heat generation at this time. The battery has a severe failure, and the state jumps directly to the serious fault state.

When the temperature consistency difference is not obvious, the difference in the heat-generation internal resistance Δ*R* is used as the characteristic parameter. The concept of heat-generation internal resistance is proposed in Feng et al. (Feng et al., 2018). Abnormal heat generation in batteries is calculated using the recursive least-squares method and expressed by introducing a variable heat-generation internal resistance. This method can eliminate the interference of heat generation in batteries when operating and the temperature inconsistency caused by the battery spatial distribution in the module. This method has a strong ability to identify faults. The specific calculation method is shown in (Feng et al., 2018). When Δ*R* is greater than 0.005 Ω, the battery exhibits self-heat generation. Since the value of Δ*R* accumulates with time, it is difficult to distinguish faults of different resistance and fault states. Therefore, heat-generation internal resistance is used only for failure judgment. The judgment of a serious fault state is realized using the abnormal d*T*_max_.

### Safety early warning strategy based on SOC consistency deviation

**Figure 9:**
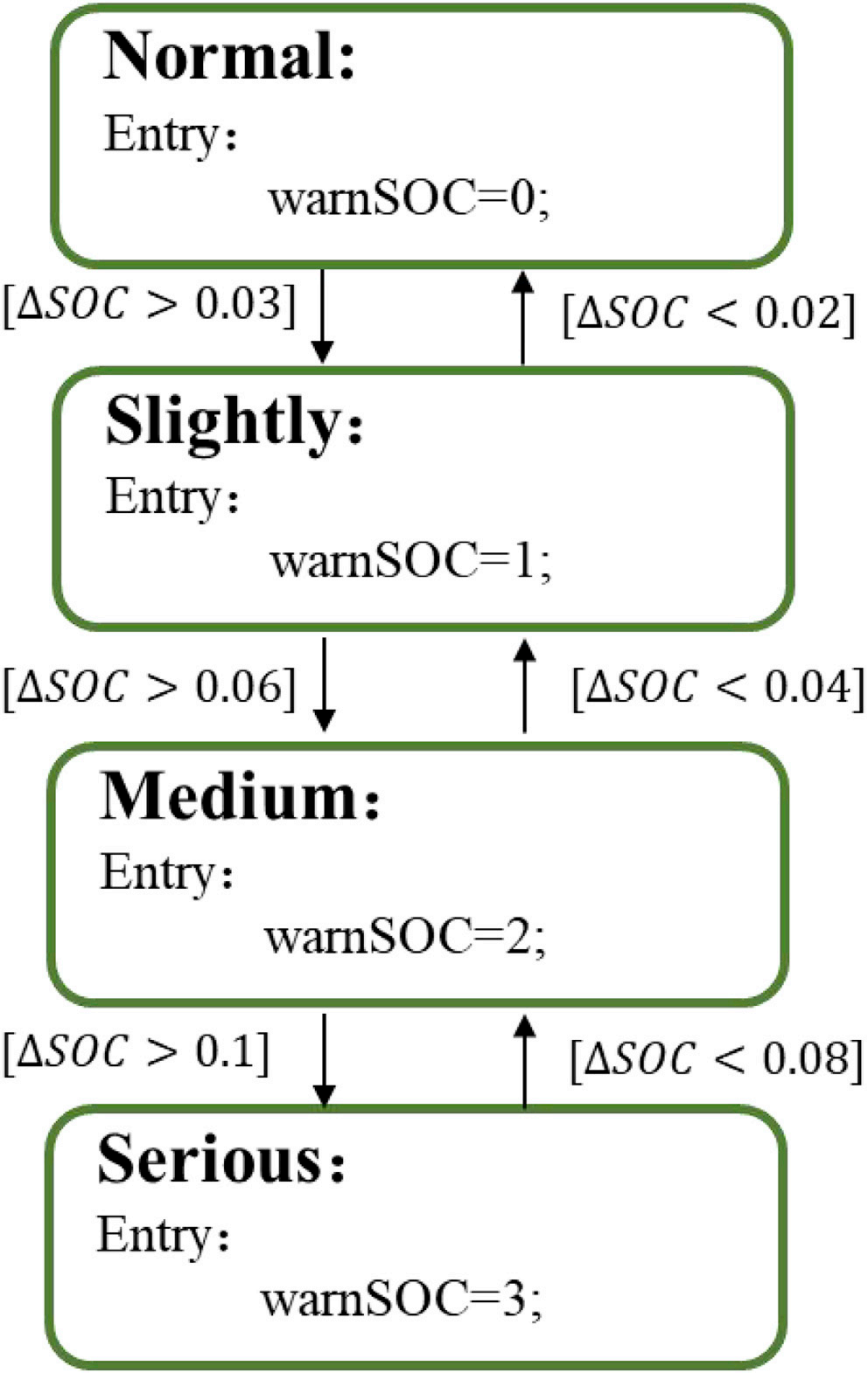
Safety early warning strategy based on SOC consistency deviation

The safety early warning strategy based on SOC consistency deviation is shown in Figure 9. The difference ΔSOC between the lowest battery SOC (SOC_min_) and the mean battery SOC (SOC_mean_) is taken as the early warning characteristic parameter. The thresholds for the transition from the normal state to slightly fault state, medium fault state, and serious fault state are set as 0.03, 0.06, and 0.1, respectively. To avoid the impact of SOC estimation error, three thresholds of 0.02, 0.04, and 0.08 are set to allow reverse state transition.

The OCV curve of LiFePO_4_ batteries has a strong nonlinear relationship due to the voltage plateau period, which makes SOC estimation more difficult and results in a higher relative error. Meanwhile, due to the short-circuit current, the measured current in the experiment is not the true value of the current flowing through the battery, interfering with the SOC estimation. To achieve higher SOC estimation accuracy, the UKF method is adopted. UKF is more suitable for nonlinear systems. It employs linear regression of multiple points collected in the prior distribution to linearize the nonlinear system equation through the unscented transformation [21]. The specific algorithm is shown in (Julier and Uhlmann, 1997). The initial values of the algorithm parameters are selected according to Equation (6), where SOC_int_ is the value of the SOC of the battery at the beginning of the working conditions.

(Equation 6) $$x_{0}=\left[\begin{array}{l} {\text{SOC}}_{\mathrm{int}}\\ 0 \end{array}\right],P_{0}=\left[\begin{array}{ll} 0.001 & 0\\ 0 & 0.001 \end{array}\right]$$

**Figure 10:**
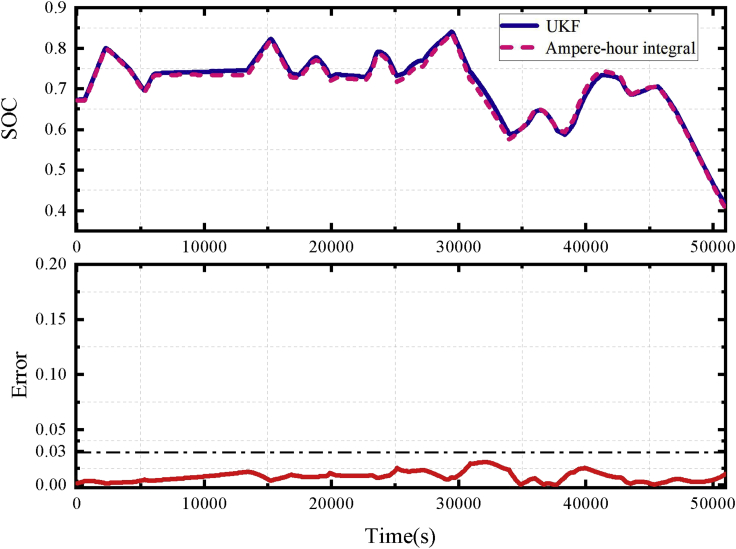
SOC estimation results under energy storage profile

The SOC estimation algorithm is verified using the SOC estimation results when the battery is operating with the profile of the energy storage system (Wang et al., 2020) under 25°C. The accurate ampere-hour integration result is used as a reference for the real SOC. The estimation result is shown in Figure 10. For normal operations, the SOC estimation has high accuracy, and the error can be within 3% with high measurement accuracy. The SOC estimation algorithm can be used to further identify the deviation of the consistency difference of the battery SOC.

### Comprehensive early warning strategy

Battery failure has different time scales and electrical and thermal characteristics. Therefore, three early warning strategies have different application ranges and response speeds. Herein, the three early warning strategies based on different characteristic parameters are combined to further analyze the battery failure. Taking the voltage, temperature, and SOC consistency deviation fault signal as 1, 2, and 3 for the slightly, medium, and serious fault states, respectively, the fault signal for a comprehensive early warning strategy can be obtained by combining the individual fault signals:

(Equation 7) $$\Phi =\Phi_{\text{Vol}}+\Phi_{\text{Temp}}+\Phi_{\text{SOC}},$$

where Φ is the fault signal of the comprehensive early warning strategy, and Φ_Vol_, Φ_Temp_, and Φ_SOC_ are the fault signals of the voltage, temperature, and SOC consistency deviation strategies, respectively. When Φ reaches 3, it is considered that a serious failure has occurred and a warning signal is issued.

## Battery fault equivalent trigger experiment of multiple timescales

The equivalent-internal-resistance fault-trigger experiment is used to simulate battery module failures with multiple time scales. This experiment simulates the failure of batteries by connecting different equivalent internal resistances to the battery and transferring the heat generated into the battery. The corresponding fault characteristics under different timescales can be obtained by changing the equivalent internal resistance value. Herein, the experiment of a single battery is first conducted to determine the appropriate equivalent internal resistance value. Then, the equivalent internal resistance value for the trigger experiment of the battery module is set, and the characteristic parameters during the battery failure are obtained. The characteristic parameters are used to verify the early warning strategies.

### Equivalent internal resistance fault-trigger experiment of single battery

**Figure 11:**
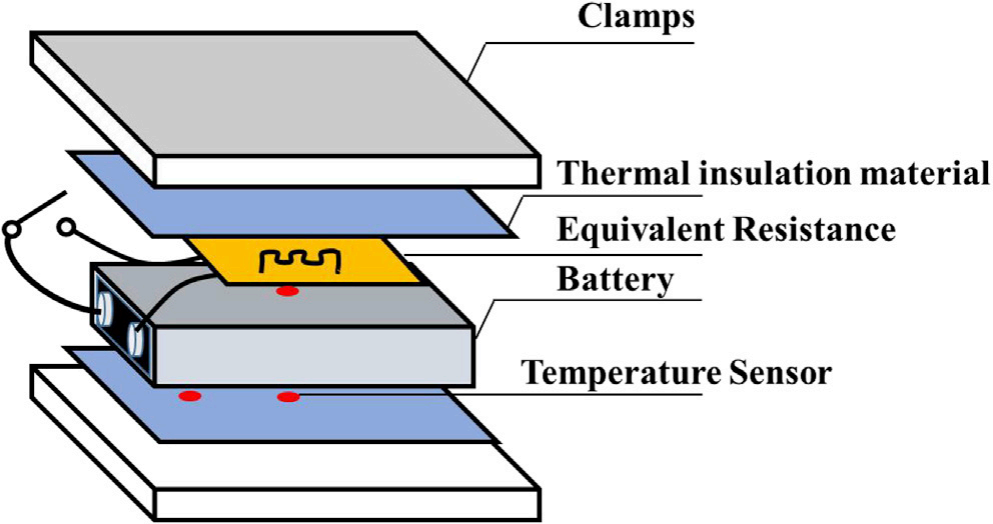
Schematic diagram of a single-battery equivalent-internal-resistance fault-trigger experiment

The method of the equivalent-internal-resistance fault-trigger experiment for a single battery is shown in Figure 11. The terminal voltage and temperature of the battery are collected by a data collector. The thermocouple used to collect the temperature signal is arranged at the equivalent internal resistance, the surface of the battery, and the end cover of the battery.

**Figure 12:**
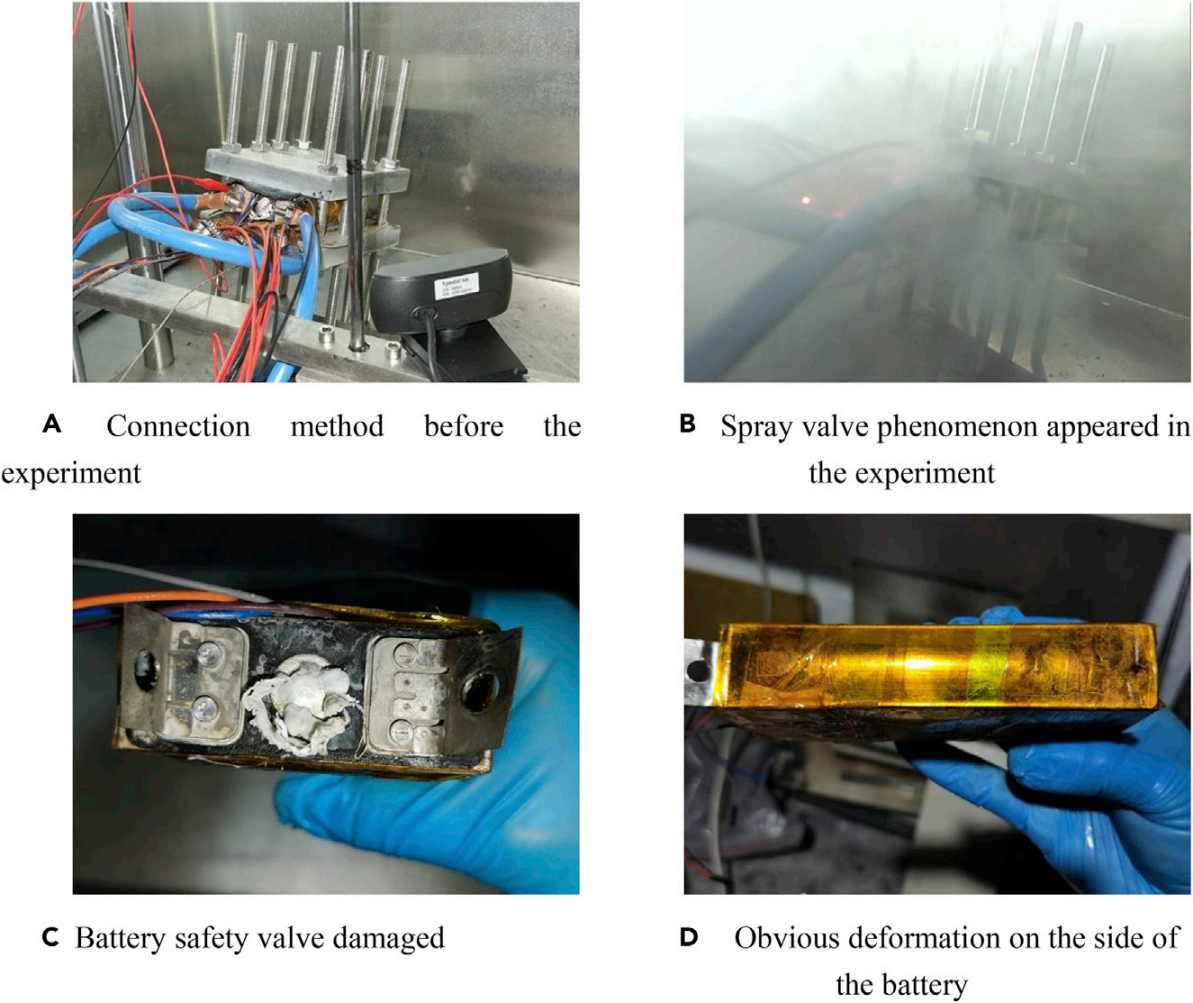
Equivalent experiment process and change in the battery morphology during 0.05-Ω equivalent internal resistance fault-trigger experiment (A) Connection method before the experiment. (B) Spray valve phenomenon appeared in the experiment. (C) Battery safety valve damaged. (D) Obvious deformation on the side of the battery.

To simulate a relatively dangerous situation, the equivalent internal resistance was gradually reduced from 0.5 Ω. It was finally chosen to be 0.5, 0.25, and 0.05 Ω. Batteries after 0.5- and 0.25-Ω experiments slightly swelled, but there was no obvious change in morphology. In the corresponding experiment for 0.05 Ω, the battery safety valve was broken and a large amount of electrolyte was sprayed out. The battery suffered a serious safety failure, as shown in Figure 12.

**Figure 13:**
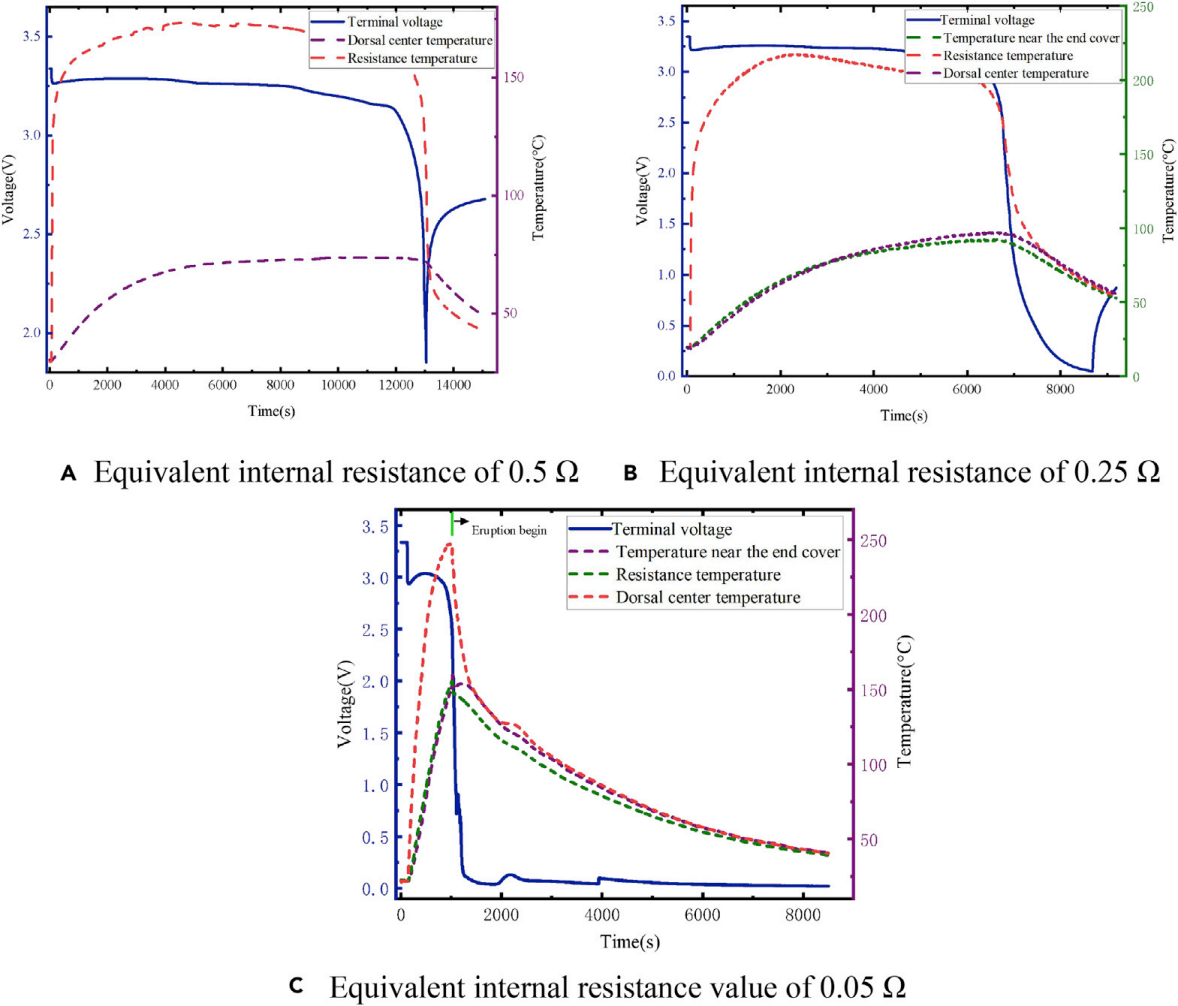
Results of equivalent internal resistance fault triggering experiments for a single battery under multi-timescale faults (A) Equivalent internal resistance of 0.5 Ω. (B) Equivalent internal resistance of 0.25 Ω. (C)Equivalent internal resistance value of 0.05 Ω.

In the three experiments with different resistance values, the experimental results of the electrical and thermal characteristics of the failed battery are shown in Figure 13. There were obvious voltage drop and abnormal temperature increase during the fault-trigger experiment. The voltage drop for the equivalent internal resistance of 0.5, 0.25, and 0.05 Ω are 37.5, 75, and 337.5 mV, respectively, and the maximum temperature of the battery surface reached 73°C, 96°C, and 153°C, respectively. According to the experiment for the single battery, the case of 20-Ah LiFePO_4_ battery failure could be classified according to the equivalent internal resistance. When the resistance was larger than 0.25 Ω, the battery temperature rise effect was not obvious, and the battery morphology did not significantly change. There were few safety risks, such as thermal runaway. When failures with an equivalent internal resistance of 0.5 Ω and below occur, the battery voltage consistency deviation is obvious and the early warning strategy relies on voltage consistency. Therefore, the module equivalent-internal-resistance fault-trigger experiments select larger equivalent internal resistance of 1 and 5 Ω for research. Since the equivalent internal resistance of 0.05 Ω responds to a relatively dangerous situation, there are high requirements for the speed of the safety-warning algorithm. Therefore, in the module equivalent-internal-resistance fault-trigger experiment, the case with the equivalent internal resistance of 0.05 Ω was studied.

### Module battery equivalent-internal-resistance fault-trigger experiment

**Figure 14:**
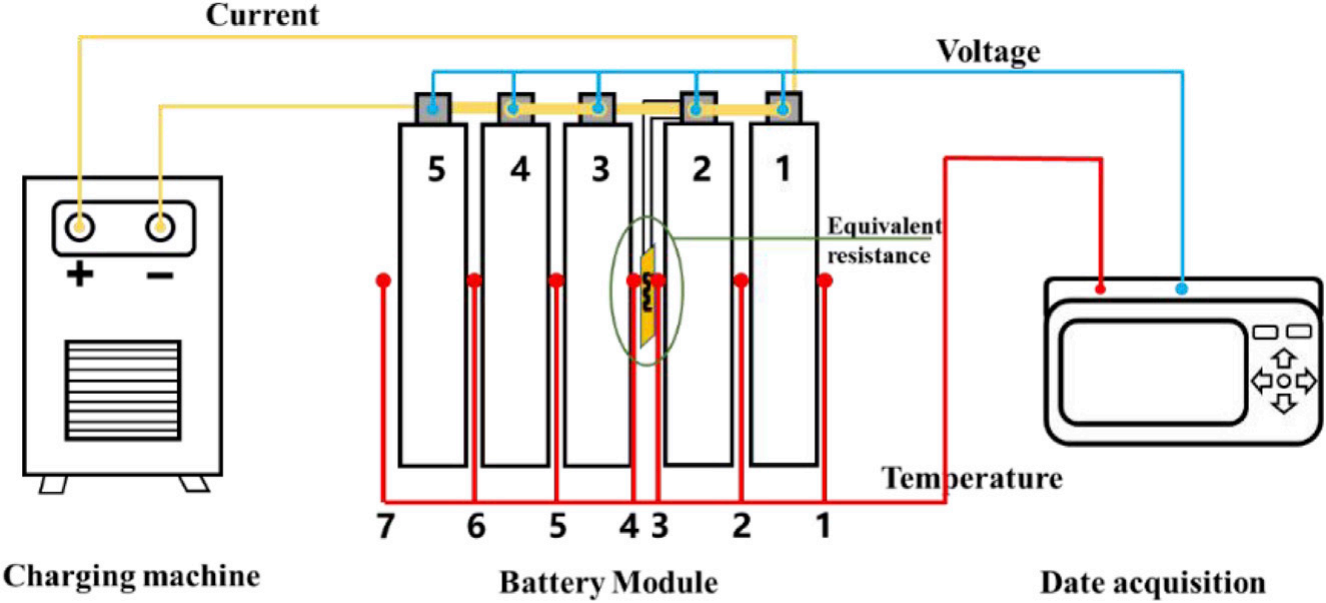
Schematic diagram of equivalent-internal-resistance fault-trigger experiment of a battery module

**Figure 15:**
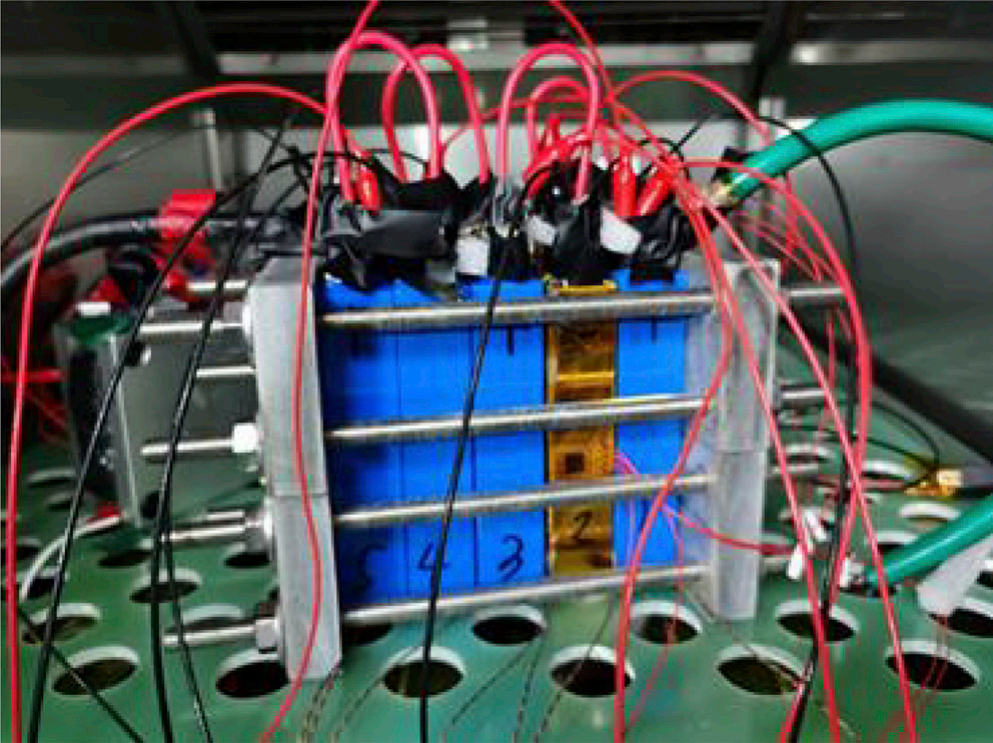
Photograph of the equivalent-internal-resistance fault triggering experiment of the modular battery

The procedure for the module battery equivalent internal resistance fault-trigger experiment is shown in Figures 14 and 15. The battery module consists of five 20-Ah LiFePO4 batteries connected in series. Battery 2 is selected as the failure battery. To meet the requirements of actual usage, a charging machine was used to run the battery under an energy storage system profile. In this experiment, the current signal was collected using the charging machine. The terminal voltage of each battery and temperature signals of the battery were collected by the data collector. Equivalent internal resistances of 0.05, 1, and 5Ω were used based on the experimental results of the single battery in Section 4.1 to simulate the fault conditions at different timescales.

**Figure 16:**
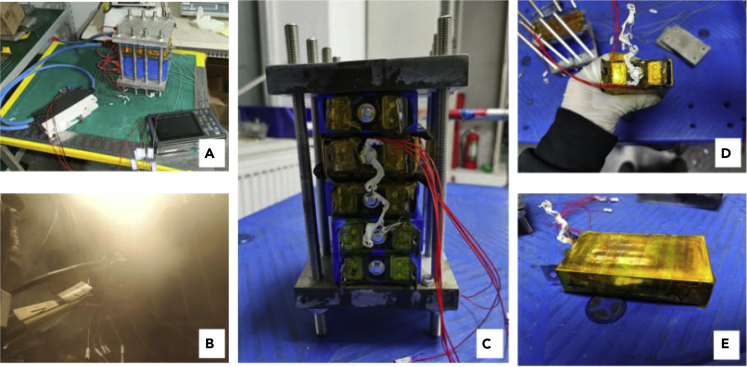
Fault triggering experiment for 0.05-Ω equivalent internal resistance fault of the single battery in the module (A) Module before the experiment. (B) Safety valve broken during the experiment. (C) Battery module after the experiment. (D) Safety valve of the failure battery was damaged. (E) Failed battery.

The results of the experiment with 0.05-Ω equivalent resistance are shown in Figure 16. The module experiment showed the same law as the single-battery experiment. After failure occurred, the temperature of the battery increased rapidly, and the battery swelled. The safety valve of the faulty battery broke 980 s after the fault began. Due to the addition of heat insulation materials, other batteries in the module were not impacted by heat conduction but only squeezed by the expansion of the faulty battery.

**Figure 17:**
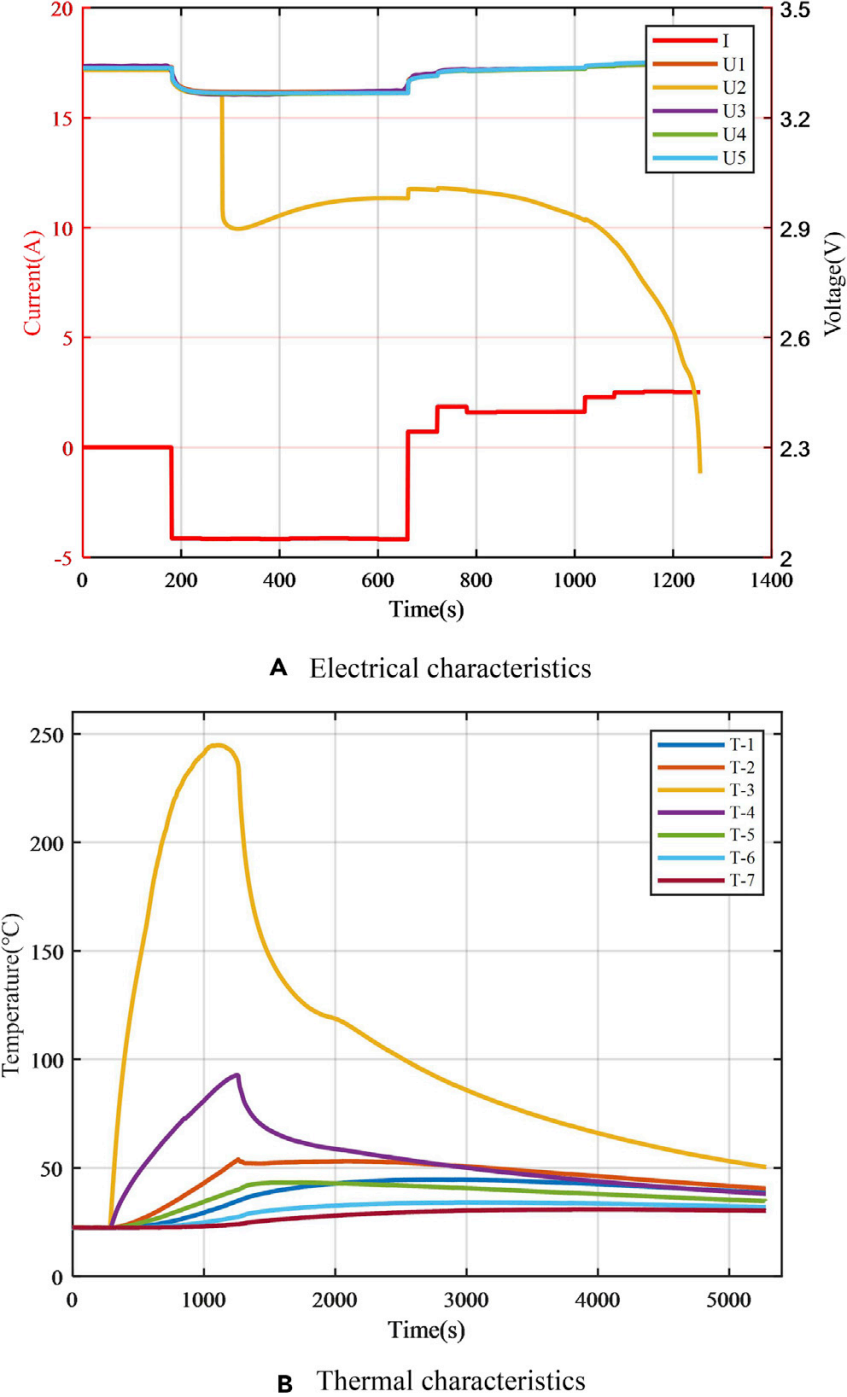
Triggering experimental results for 0.05-Ω equivalent internal resistance (A) Electrical characteristics. (B) Thermal characteristics.

The results of the electrical and thermal characteristics of the battery are shown in Figure 17, where *I* represents the operating current of the battery module, *U*_1_*–U*_5_ are the terminal voltages of the five battery cells in the module, and *T*_1_–*T*_7_ are temperature signals of seven temperature sensors arranged. *T*_3_ and *T*_4_ are the temperatures on the equivalent internal resistance, which are difficult to detect in actual situations and are mostly ignored. Figure 17A shows that since the equivalent internal resistance was relatively small, the current flowing through the equivalent internal resistance was high and the voltage of the failed battery was different from that of other batteries in the module. Early warning strategies based on voltage consistency deviation showed a good effect. The temperature of the failed battery was also significantly different from that of other batteries in the module (Figure 17B). However, heat transfer and different heat dissipation conditions of the batteries in the module resulted in inconsistency of temperature between the batteries in the module, and this affects the early warning algorithm.

**Figure 18:**
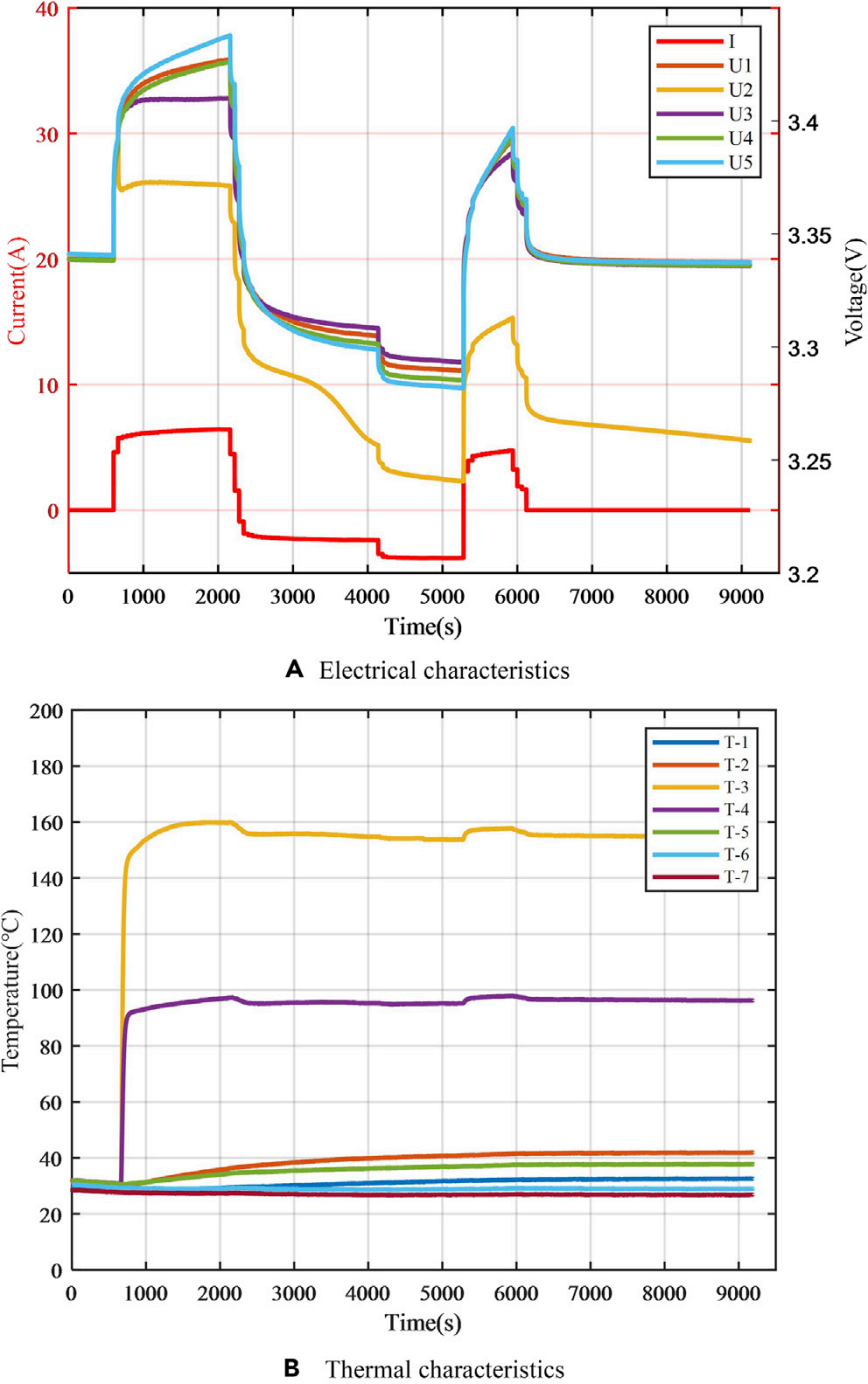
Triggering experiment results for 1-Ω equivalent internal resistance (A) Electrical characteristics. (B) Thermal characteristics.

The result of the fault-trigger experiment with an equivalent internal resistance of 1 Ω is shown in Figure 18. The terminal voltage of the failed battery was significantly lower than that of other batteries. However, the temperatures of the batteries near the faulty battery were also affected by heat conduction, reducing the voltage and temperature consistency of the battery pack. This causes certain interference to the safety early warning strategy based on the voltage consistency deviation. In this case, the strategies based on voltage and temperature consistency deviations are applicable. The speed of early warning is better than the strategy based on the SOC consistency deviation.

**Figure 19:**
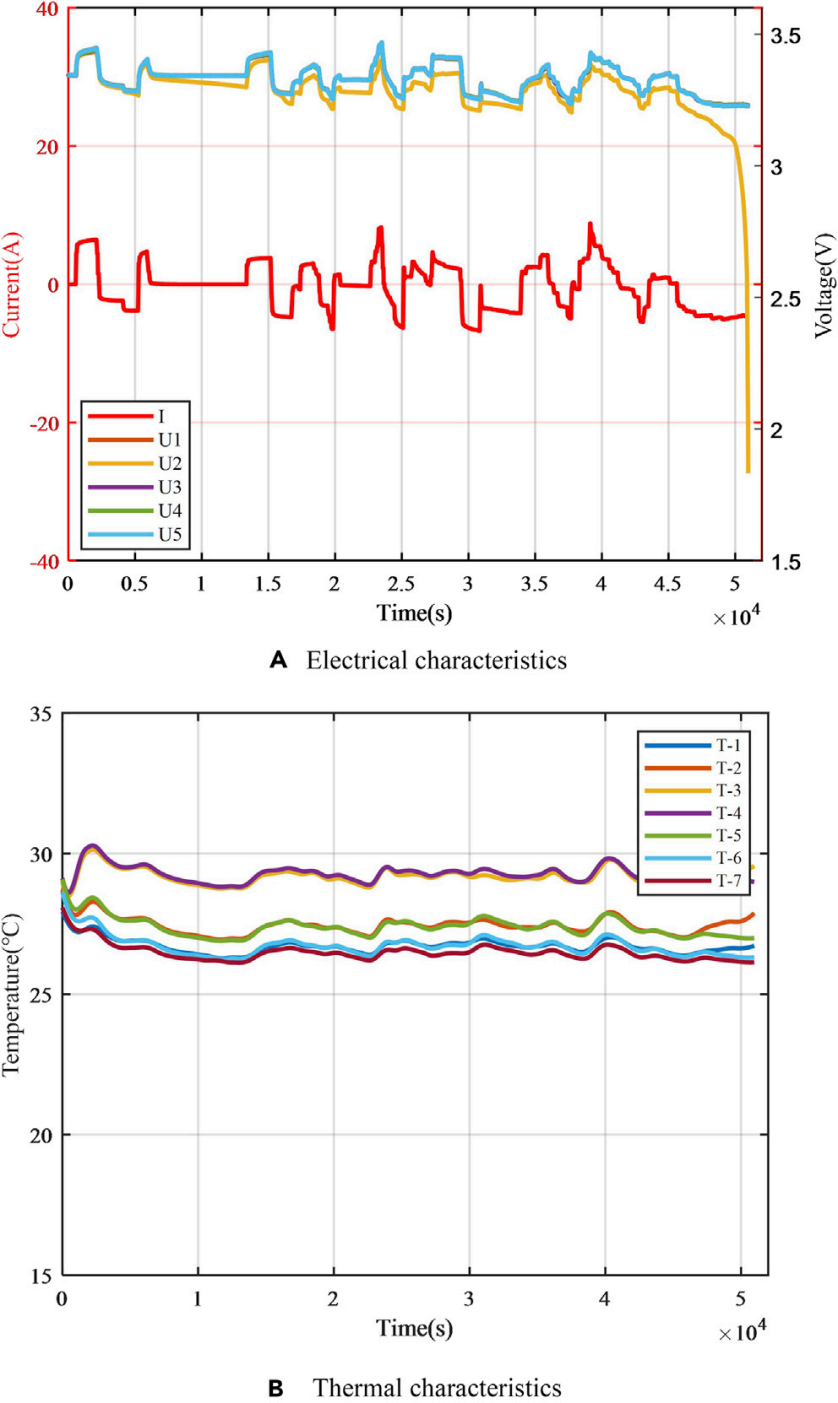
Triggering experimental results for 5-Ω equivalent internal resistance (A) Electrical characteristics. (B) Thermal characteristics.

The results of the 5-Ω equivalent-internal-resistance fault triggering experiment are shown in Figure 19. After 656 s of triggering the fault, the voltage difference of the battery was not obvious until the SOC consistency deviation of the batteries was high due to self-discharge. The voltage difference slightly increased due to the difference in the OCV of the battery. There was only a small voltage difference between the faulty and other batteries in the whole process because the OCV of the LiFePO_4_ battery exhibits a very flat voltage plateau. The voltage difference was small until the SOC of the faulty battery was close to 0%. Then, the voltage difference of the battery became obvious. The temperature measurement results show that in addition to the high inconsistency in the temperature near the equivalent internal resistance, the temperature difference among other batteries was small. It is difficult to distinguish temperature differences from the uneven distribution of temperature due to the spatial distribution of batteries. Early warning strategy relies more on the strategy of SOC consistency deviation.

## Validation and analysis of the early warning strategy

The comprehensive early warning strategy was verified using the results of the module equivalent-internal-resistance fault-trigger experiment. Herein, comprehensive early warning algorithms based on the consistency deviation of voltage, temperature, and SOC were developed. Three fault conditions of different time scales were verified.

### Early warning effect with the equivalent resistance of 5 Ω

**Figure 20:**
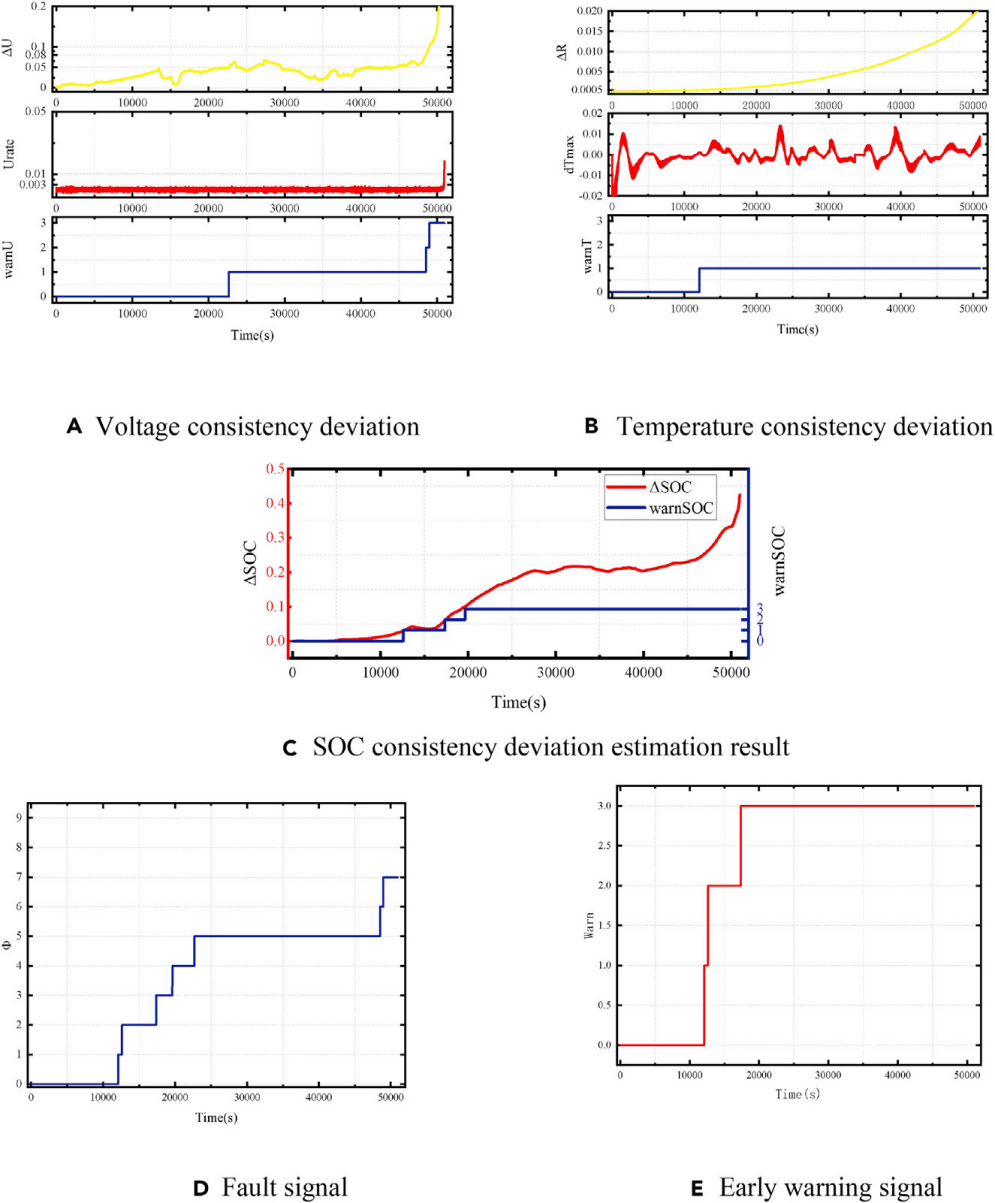
Early warning results of a 5-Ω equivalent-internal-resistance failure of the battery module (A) Voltage consistency deviation. (B) Temperature consistency deviation. (C) SOC consistency deviation estimation result. (D) Fault signal. (E) Early warning signal.

The consistency deviation of the battery, fault position signal, and early warning signal results are shown in Figure 20. The equivalent internal resistance is 5 Ω.

The fault was triggered at t = 659 s. Due to the relatively high equivalent internal resistance, the corresponding scenario is battery failure with a long timescale. The strategy based on estimating the voltage consistency deviation needs to consider the consistency of the battery and the influence of the sampling noise. Therefore, the threshold of the early warning strategy should not be too low. It is difficult to achieve rapid warning, in this case, using the voltage consistency strategy. The strategy based on voltage consistency deviation can detect obvious voltage abnormalities about 20,000 s after a fault occurs. Meanwhile, the voltage platform of LiFePO_4_ batteries makes the terminal voltage of the battery change a little during the short-circuit process. The battery would have a significant voltage difference only when it is close to the end of the discharge. An alarm signal can be issued. Temperature consistency and SOC deviations are parameters accumulated over time. They can be used for fault diagnosis with higher resistance. However, the battery temperature does not obviously increase with high equivalent resistance. Although the early warning strategy based on heat-generation internal resistance can detect the difference in battery self-heating, it cannot identify the fault level. In this case, the early warning strategy based on the consistency deviation of SOC is more applicable. It can quickly identify the occurrence of a fault and further predict the severity of the fault. Finally, the strategy based on SOC consistency deviation can realize early warning for the serious fault conditions.

### Early warning effect with the equivalent resistance of 1 Ω

**Figure 21:**
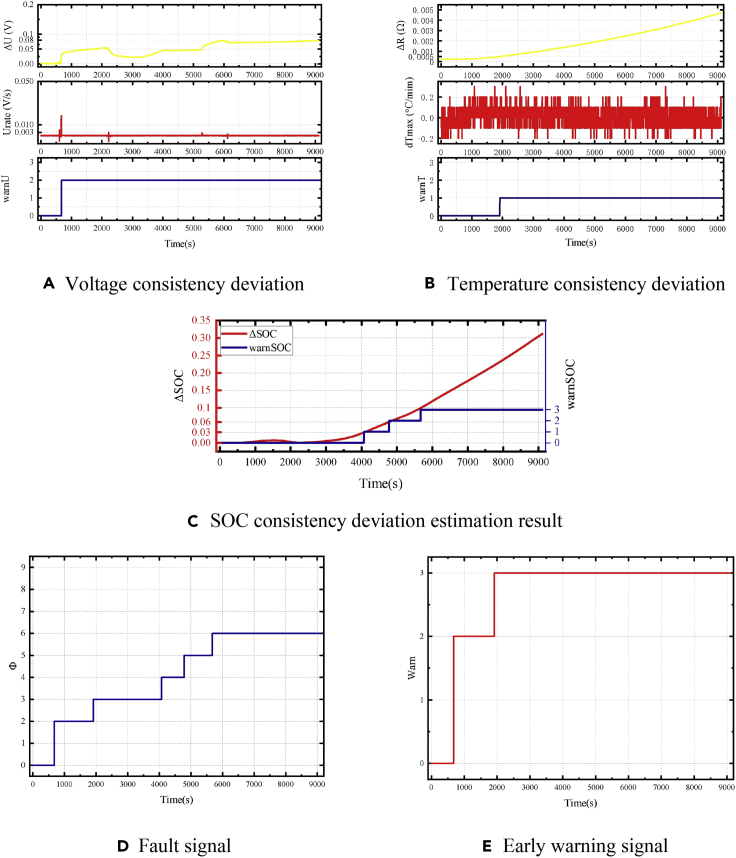
Early warning results of a 1-Ω equivalent internal resistance failure of the battery module (A) Voltage consistency deviation. (B) Temperature consistency deviation. (C) SOC consistency deviation estimation result. (D) Fault signal. (E) Early warning signal.

For a fault occurring with an equivalent internal resistance of 1 Ω, the consistency calculation result of the battery, the fault bit signal, and the early warning signal is shown in Figure 21. The fault in the battery is triggered after 665 s, and it corresponds to a fault situation of the middle timescale. When the fault is triggered, the early warning strategy based on the *U*_*rate*_ of the battery detects the obvious abnormalities in the voltage difference consistency and outputs a fault signal Φ_Vol_ = 2. Herein, after 1911 s, the temperature warning signal showed an abnormality. However, the battery voltage and temperature consistency deviations did not reach the level of serious failure at this time. With the accumulation of time and an increase in battery self-discharge, the early warning strategy based on estimating the SOC consistency deviation detected an abnormality at 4,342 s.

### Equivalent internal resistance of 0.05Ω

**Figure 22:**
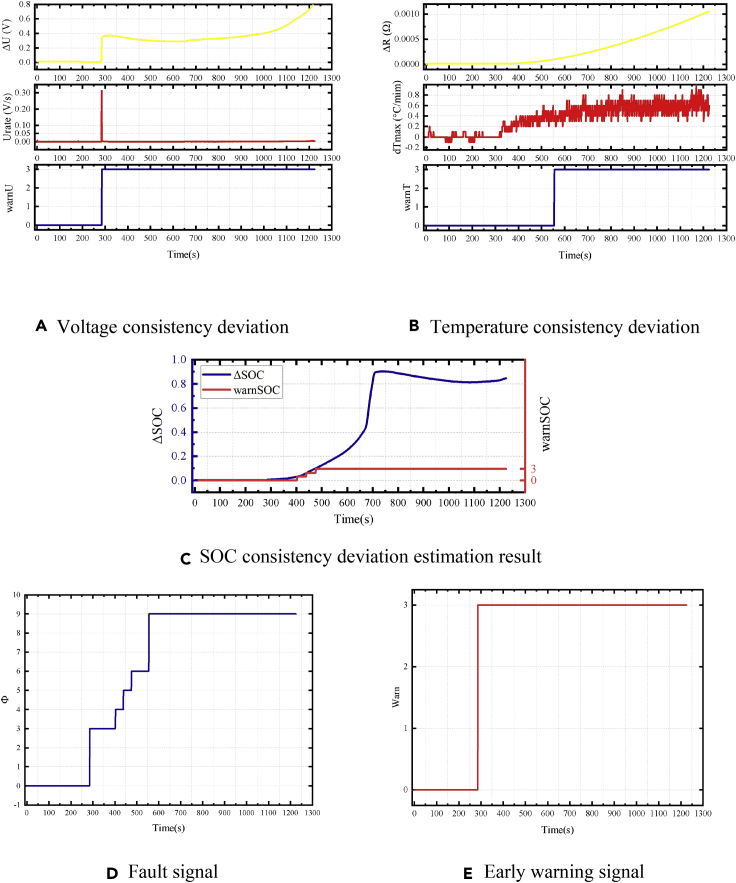
Early warning results of 0.05-Ω equivalent-internal-resistance failure of the battery module (A) Voltage consistency deviation. (B) Temperature consistency deviation. (C) SOC consistency deviation estimation result. (D) Fault signal. (E) Early warning signal.

The result of the early warning strategy when the battery failed with an equivalent internal resistance of 0.05 Ω is shown in Figure 22. The fault occurred at 284 s. Since the equivalent internal resistance was small, the corresponding fault was a short timescale. At this time, the voltage consistency deviation of the battery and the deviation *U*_*rate*_ instantly increased, and the voltage early warning strategy immediately determined that the battery has a serious failure. Meanwhile, the parameters of the SOC estimation algorithm were updated according to the results of the early warning strategy of the voltage consistency deviation. The fault signal of the early warning strategy based on SOC also reached the serious fault level within 500 s. An extremely high rate of self-heat generation of the battery was also detected by the temperature consistency warning algorithm, and the temperature consistency strategy also reached a serious failure state. In this case, the battery safety valve eventually breaks, and a local thermal runaway occurs. It is a dangerous situation with higher requirements for early warning speed. The battery safety valve broke 980 s after the failure, and the early warning strategy could detect a serious fault 900 s before more dangerous security issues arose.

### Analysis and comparison of early warning strategies

**Figure 23:**
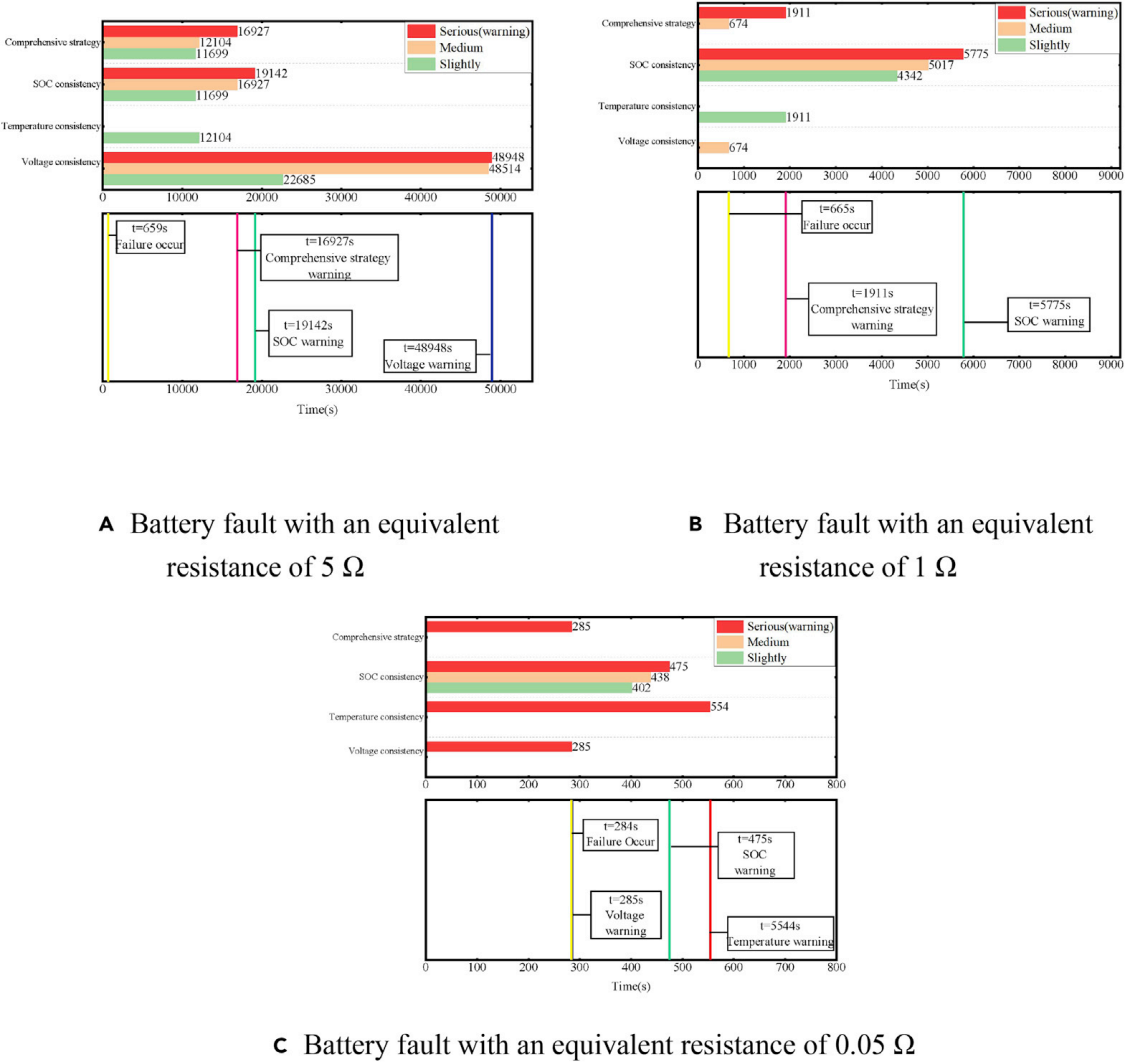
Effect of early warning strategies with different equivalent resistances (A) Battery fault with an equivalent resistance of 5 Ω. (B) Battery fault with an equivalent resistance of 1 Ω. (C) Battery fault with an equivalent resistance of 0.05 Ω.

To show the effects of different early warning strategies more clearly, the early warning effects of three timescales of the fault situation were analyzed, as shown in Figure 23. The warning time of the three early warning strategies and the comprehensive early warning strategy identifying different levels of faults were plotted. The time it takes for each strategy to achieve an alarm individually, i.e., Φ, Φ_Vol_, Φ_Temp_, or ΦSOC reaching the serious fault state, is compared.

For long-timescale situations with an equivalent resistance of 5 Ω, the resistance is relatively high and the effect of early warning strategy based on voltage consistency deviation is not obvious. Early warning strategy based on voltage consistency deviation takes more than 48,000 s to detect a fault. Although the strategy based on the estimation of temperature consistency deviation can quickly detect failures, it is difficult to distinguish between different fault levels because of the difference in the heat-generation internal resistance. Temperature is a relatively indirect parameter, which is affected by environmental conditions and the spatial distribution of batteries in a module. The early warning strategy based on the estimation of SOC consistency deviation has a good effect. Although the estimated SOC of the failed battery slightly lags behind the actual battery SOC, the early warning time based on the SOC consistency deviation is still better than that of the other two strategies. The strategy based on comprehensive early warning surpasses the SOC and temperature consistency deviation strategy. Compared with the strategy based on the deviation of SOC, the comprehensive early warning strategy can diagnose a fault 2000 s earlier. The comprehensive early warning strategy can realize early warning with a speed of hour level.

For a medium-timescale situation with an equivalent internal resistance of 1 Ω, the voltage consistency deviation strategy has a good effect. The early warning strategy based on *U*_*rate*_ gives a fault signal 9 s after the fault occurs with a fault level of 2. The early warning strategy based on temperature consistency deviation gives an early warning signal 1,246 s after the failure occurs, and the comprehensive early warning strategy achieved early warning. The early warning strategy based on the consistency deviation of SOC has a slower speed than the voltage and temperature consistency deviation strategies, although the parameters of the SOC estimation algorithm are adjusted according to the early warning strategy based on voltage consistency deviation. In general, the proposed comprehensive early warning strategy can achieve minute-level early warning for medium timescale failures.

For a short timescale situation of equivalent internal resistance of 0.05 Ω, the early warning strategy based on voltage consistency deviation can send a warning signal within 1 s through a fast-rising path. The early warning strategy based on temperature consistency can also detect the abnormal rate of temperature rise 270 s after the fault occurs. According to the results of the strategy based on the consistency of *U*_*rate*_, the parameters of the SOC estimation algorithm are adjusted. At this time, the early warning strategy based on SOC can reach the fault signal level of 3 at 191 s after the fault occurs and sends a warning signal. For serious faults on a short timescale, the comprehensive early warning strategy can achieve a second-level early warning, sending a warning signal 900 s before the serious failure of the battery results in a local thermal runaway and the safety valve breaks.

In general, for faults with small equivalent internal resistance and short timescales, the voltage difference is high and the impact of sampling error is small. The early warning strategy based on voltage consistency deviation, thus, has a good effect. The early warning algorithm based on SOC consistency deviation can further determine the fault level after the voltage warning, and that based on temperature consistency deviation can assist other warning strategies to achieve a higher warning speed. When the equivalent internal resistance is extremely small, the strategy based on temperature consistency deviation can detect obvious self-generated heat in the faulty battery and realize early warning independently. For the case of high equivalent internal resistance and long timescales, the strategies based on voltage and temperature consistency deviations are ineffective due to the influence of the voltage platform on LiFePO_4_ batteries. Then, the strategy based on SOC consistency has a better effect. A comprehensive early warning strategy, which varies from the temperature and SOC consistencies can effectively improve the early warning speed.

### Conclusion

At present, there has been some progress in the study of thermal runaway mechanism and internal short circuit identification methods, but the research objects are mostly automotive scenarios and ternary Li-ion batteries. Research on lithium iron phosphate batteries and energy storage scenarios is not yet in-depth, in order to solve the above problems. We developed a comprehensive early warning strategy for multiple timescales of consistent deviation estimation of electric and thermal characteristics to solve the problem of safety early warning in LiFePO_4_ batteries used in energy storage systems. The electric and thermal characteristics of a LiFePO_4_ battery module under different time scales were obtained through module equivalent-internal-resistance fault-trigger experiments. The experimental results prove the effectiveness of the comprehensive early warning strategy. Based on the results, the following conclusions are drawn:

(1) The proposed comprehensive early warning strategy combines a short timescale strategy based on voltage consistency deviation and a long-timescale early warning strategy based on SOC consistency deviation. It expands the scope of applications of early warning algorithms. According to the results of the module equivalent-internal-resistance fault-trigger experiments, the strategy can realize the early warning at different timescales. For the short timescale situation of 0.05 Ω equivalent internal resistance, it can give an early warning signal 15 min in advance.
(2) The UKF algorithm was used to estimate the SOC of the faulty battery because the flat charge/discharge voltage curve of LiFePO4 battery, If the short-circuit current cannot be detected when the fault occurs, the SOC estimation algorithm can still obtain the SOC of the faulty battery. At this time, the estimation error is relatively high, but the early warning of battery failure can be realized through the difference in SOC consistency of different batteries in the module.

### Limitations of the study

The early warning strategy studied in this paper is based on the estimation and measurement of thermoelectric parameters of energy storage battery, which is highly dependent on the state estimation accuracy of energy storage battery.

## STAR★Methods

### Key resources table

**Table:** 

REAGENT or RESOURCE	SOURCE	IDENTIFIER
**Critical commercial assays**		

Battery	Lishen	LP2770134-20Ah

**Software and algorithms**		

Origin2020	Originlab	http://www.OriginLab.com
BTS 600	Digatron	https://www.digatron.com/en-us/Software

### Resource availability

#### Lead contact

Further requests for information should be directed and will be handled by the corresponding author and lead contact, Jiuyu Du (dujiuyu@tsinghua.edu.cn).

#### Materials availability

The study did not generate new materials.

### Methods details

#### Experimental battery parameters

The nominal capacity of the LiFePO_4_ battery used in this research was 20 Ah with the nominal voltage of 3.2 V. The average measured capacity and mass are 21.8 Ah and 515 g, respectively.

#### Experiment scene setting

In order to ensure the insulation between the batteries during the experiments, the batteries were covered with heat shrinkable film in advance. To emulate realistic scenarios and prevent heat loss, mica sheets are placed on both sides of the module and tightened with screws, the 0.05 Ω resistance used in the triggering experiment was made of hollow silicon steel sheets arranged in a snake-like arrangement to match the size of the battery. Resistance wire is selected in another triggering experiments, precision resistance tester is chosen as the experiment for measuring resistance, after selecting the desired length of resistance wire, it is also bent into a snake shape, with high temperature resistant tape fixed, to prevent the resistance wire contact caused by short circuit, this short circuit will lead to the realistic resistance is smaller than the measurement resistance.

#### Equipment used in the experiment

The charge/discharge machine used in this paper is Digatron BTS-600; The data acquisition device is HIOKI LR8450. During the experiments, the temperature was set at 25°C.

## Data Availability

The data generated in this study could not be shared due to confidentiality.
